# Auxins and Cytokinins in Plant Development 2018

**DOI:** 10.3390/ijms20040909

**Published:** 2019-02-20

**Authors:** Jan Petrasek, Klara Hoyerova, Vaclav Motyka, Jan Hejatko, Petre Dobrev, Miroslav Kaminek, Radomira Vankova

**Affiliations:** 1 Laboratory of Hormonal Regulations in Plants, Institute of Experimental Botany, The Czech Academy of Sciences, Rozvojova 263, 16502 Prague 6, Czech Republic; petrasek@ueb.cas.cz (J.P.); hoyerova@ueb.cas.cz (K.H.); motyka@ueb.cas.cz (V.M.); dobrev@ueb.cas.cz (P.D.); kaminek@ueb.cas.cz (M.K.); 2 CEITEC–Central European Institute of Technology and Functional Genomics and Proteomics, NCBR, Faculty of Science, Masaryk University, 62500 Brno, Czech Republic; hejatko@sci.muni.cz

**Keywords:** auxin, cytokinin, plant development, plant hormone crosstalk

## Abstract

The international symposium “Auxins and Cytokinins in Plant Development” (ACPD), which is held every 4–5 years in Prague, Czech Republic, is a meeting of scientists interested in the elucidation of the action of two important plant hormones—auxins and cytokinins. It is organized by a group of researchers from the Laboratory of Hormonal Regulations in Plants at the Institute of Experimental Botany, the Czech Academy of Sciences. The symposia already have a long tradition, having started in 1972. Thanks to the central role of auxins and cytokinins in plant development, the ACPD 2018 symposium was again attended by numerous experts who presented their results in the opening, two plenary lectures, and six regular sessions, including two poster sessions. Due to the open character of the research community, which is traditionally very well displayed during the meeting, a lot of unpublished data were presented and discussed. In this report, we summarize the contributions in individual sessions that attracted our attention.

## 1. Introduction

The international symposium “Auxins and Cytokinins in Plant Development... and Interactions with Other Phytohormones 2018” (ACDP 2018) was held on 1–5 July in Prague, the Czech Republic (https://www.acpd2018.org/). The meeting was attended by 233 participants from 28 countries. This symposium was the ninth in the series of hormone-related symposia organized by the Institute of Experimental Botany, the Czech Academy of Sciences. The first four conferences, held in Liblice castle, covered all hormones, which reflected the early stage of phytohormone studies. Beginning in 1999, the symposia were moved to Prague and focused on auxins and cytokinins (named “Auxins and Cytokinins in Plant Development”) as vast progress in the research of these two important classes of hormones had been achieved. Later on, elucidation of a complex hormonal network based on intensive interactions among different hormones imposed a requirement for extension of the symposium focus to interactions of auxins and cytokinins with other phytohormones.

Since the very beginning, true legends of the phytohormone field like Folke Skoog, Kenneth V. Thimann, Carlos O. Miller, and David and Machteld Mok participated in setting up the high scientific standard of the symposia. Even though the generations of researchers have changed over time, ACPD has still remained a prestigious meeting focused on breakthrough findings in the field. The communication among the generations that built the basis of hormonal research and the young researchers, that follow, provided depth for scientific discussions and building new hypotheses. The symposia have provided an opportunity to present the current state of knowledge in hormone research and have enabled lively discussions on new achievements, strengthening existing and establishing new cooperation.

## 2. Symposium Opening and Plenary Lectures

The program of ACPD2018 started with an opening lecture and two plenary ones. Then, six sessions consisting of oral and poster presentations followed: (1) Biosynthesis and metabolism, (2) Signalling, (3) Development, (4) Transport, (5) Interactions and crosstalk, and (6) Interaction with the environment. 

The opening lecture was given by Dirk Inze (VIB-UGent, Belgium), who outlined the molecular network regulating plant organ growth. He demonstrated that developmental programs are modulated by the actual environmental conditions and stressed the pivotal role of auxins and cytokinins in the determination of organ formation and size. He showed the phenotype of plants overexpressing growth-enhancing gene, which represents a promising strategy for crop improvement. Joe Kieber (University of North Carolina, USA) described in his plenary lecture the cytokinin-regulated transcriptional network. He outlined new directions in cytokinin actions. It seems that cytokinins are able to control chromatin accessibility, making the DNA more “opened”. The enrichment of type-B Response Regulator (RR) target sites in those regions implies a possible role of these RRs in cytokinin-dependent chromatin remodelling. The new role of cytokinins in the control of autophagy as a possible mechanism of cytokinin-regulated senescence was suggested via control of the protein stability of type-A RRs by paralogues of exocyst subunit EXO70D. Jiří Friml (IST, Austria) summarized how our knowledge of auxin-regulated development has advanced during the past two decades and further described the mechanisms of PIN protein polarizations during early embryogenesis, hypocotyl bending, and vascular tissue development. The role of transcription factor WRKY23 in the coordination of the transcriptional network upstream of PIN polarization was shown to be potentially crucial for auxin canalization-driven processes.

## 3. Biosynthesis and Metabolism

This session encompassed nine oral presentations pointing mostly at metabolic pathways and mechanisms controlling auxin and cytokinin homeostasis and its regulation. “Keeping the balance” was a motto of the first talk, by Karin Ljung (Umeå University, Sweden). She presented interesting data on the transcriptional control of auxin biosynthesis by cytokinin signalling systems, thus suggesting possible crosstalk of both hormone groups for fine-tuning of their metabolic levels. Ondřej Novák (Palacký University & IEB CAS, Czech Republic) demonstrated new findings towards the localization of auxin and cytokinin derivatives on cellular and subcellular levels by novel analytical tools combining different approaches such as density gradient ultracentrifugation or fluorescence-assisted cell/organelle sorting (FACS/FAOS) with micropurification protocols and ultra-sensitive mass spectrometry techniques. David Kopečný (CRH & Palacký University, Czech Republic) presented a new generation of highly specific cytokinin oxidase/dehydrogenase inhibitors derived from thidiazuron and phenylureas and outlined their potential as attractive candidates for in planta trials. Federica Brunoni (Umeå University, Sweden) reported on 2-oxoglutarate-dependent-Fe(II) dioxygenase enzymes coded by DIOXYGENASE FOR AUXIN OXIDATION (DAO) genes, which catalyse the formation of the primary indole-3-acetic acid (IAA) catabolic product 2-oxindole-3-acetic acid (oxIAA). Hitoshi Sakakibara (Nagoya University, Japan) outlined recent progress in cytokinin research and summarized cytokinin metabolic pathways and their regulatory steps. The different roles of hydroxylated versus nonhydroxylated cytokinins, i.e., *trans*-zeatin (tZ) vs. *N*^6^-(Δ^2^-isopentenyl)adenine (iP) types, their site of production, and their transport were shown in distinct plant organs such as shoots and roots. Petr Hošek (IEB CAS, Czech Republic) reported on the construction and optimization of a computational model providing estimates of the kinetic rates of major cytokinin metabolic reactions. Ioanna Antoniadi (Umeå University, Sweden) presented results on an *ipt29* double knockout mutant revealing that the cytokinin biosynthetic enzymes, IPT2 and/or IPT9, affected not only cytokinin production but also auxin metabolism and, subsequently, root growth. Ibraheem Alimi (Trent University, Canada) demonstrated the ability of the fungus *Ustilago maydis* to secrete active cytokinins which promoted excessive cell division in the host *Zea mays* tissue associated with the formation of tumours. The last talk of the session, given by Oh Man-Ho (Chungnam National University, Republic of Korea), was focused on the regulation of glucosinolate formation in *Arabidopsis* and radish by brassinosteroids.

## 4. Signalling

This session included 15 oral presentations focused on individual features of auxin and cytokinin signal transduction with respect to species specificity. The presentations also included an elucidation of the hormonal regulations involved in the control of cambial activity. Mark Estelle (University of California, USA) summarized the state of the art of auxin signalling, especially the complexity of Aux/IAA and ARF (Auxin Response Factor) interactions. The biggest challenge has been to address the mechanisms underlying the dual role of ARFs in activating and repressing downstream genes and to understand the role of the evolutionary old chromatin association of some AFBs (Auxin Receptor F-box proteins). Richard Napier (University of Warwick, UK) described how influential the mapping of the selectivity of auxin receptors from the TIR1/AFBs family might be towards numerous auxin herbicide scaffolds, also using models of *Marchantia polymorpha* and *Physcomitrella patens*. He pointed to the ancestrally conserved selectivity of TIR1 (TRANSPORT INHIBITOR RESPONSE1) and also identified a novel auxin compound, indole-3-methyl tetrazole, that binds selectively to TIR1 but not to AFB5. Matyáš Fendrych (IST, Austria) reported on the auxin role in root growth elongation, including gravitropic bending. The very fast response involves a TIR1/AFB-Aux/IAA co-receptor complex, but not downstream transcriptional reprogramming, which hints to a novel, non-transcriptional branch of the auxin signalling pathway. Hartwig Luethen (University of Hamburg, Germany) introduced his models of swelling protoplasts and imaging-based auxanometers for recording rapid growth responses in excised hypocotyls and intact roots of *Arabidopsis* seedlings. These methods were used to discriminate between ABP1- and TIR1/AFB-mediated processes in *Arabidopsis*. ABP1 seems to be involved in mediating rapid auxin-induced protoplast swelling, but it is not involved in the control of auxin-induced rapid growth. Charo Del Genio (University of Warwick, UK) introduced a modelling approach for the determination of the molecular dynamics of auxin binding to TIR1 receptor. This approach allowed the uncovering of ligand-induced TIR1 structural changes, electrostatic locking of auxin molecules on specific amino acid residues, and the role of this stabilization in subsequent degron binding. Thomas Schmülling (Freie Universität Berlin, Germany) focused on the elucidation of cytokinins’ role in stress responses, particularly in drought. He demonstrated at least partial transferability of the findings from *Arabidopsis* (e.g., improved drought resistance of CYTOKININ OXIDASE/DEHYDROGENASE overexpressing lines) to crops (barley and/or oil-seed rape). Ildoo Hwang (Pohang University of Science and Technology, Republic of Korea) presented a comprehensive study on BIN2-LIKE 1 (BIL1), a member of the glycogen synthase kinase 3 family, which integrates the role of auxin and cytokinin signalling into the regulation of secondary growth. BIL1 is able to phosphorylate MP (MONOPTEROS)/ARF5, leading to the up-regulation of type-A RRs (ARR7 and ARR15) and resulting in the attenuation of cytokinin signalling and down-regulation of cambial activity. On the other hand, PXY/TDR (PHLOEM INTERCALATED WITH XYLEM/TRACHEARY ELEMENT DIFFERENTIATION INHIBITORY FACTOR RECEPTOR) inhibits BIL1 activity, thus mediating the up-regulation of cambial activity. Jan Hejátko (CEITEC, Masaryk University, Czech Republic) presented data on the structure of AHP2 (ARABIDOPSIS HISTIDINE-CONTAINING PHOSPHOTRANSMITTER), which acts downstream of cytokinin receptors, including sensor kinase CKI1. He demonstrated a novel mechanism determining structure-driven signalling specificity of the plant multistep phosphorelay pathway. Sergey Lomin (Timiryazev Institute of Plant Physiology, RAS, Russian Federation) presented the identification and intracellular functioning of potato cytokinin receptors. The expression of potato cytokinin receptors was found to be organ-specific and sensitive to growth conditions, particularly to sucrose content. Using a phylogenomic approach, Dolf Weijers (Wageningen University, Netherlands) presented recent data on the origin and evolution of nuclear auxin response machinery. He described the role of evolutionary ancient transcription factors, “proto-ARFs“, and their potential function in auxin signalling. Jekaterina Truskina (ENS Lyon, France) described the identification of the upstream activators of five ARFs. The network of tissue-specific regulators was shown, with the majority represented by novel repressors of ARF transcription. Tongda Xu (Fuijan University, China) described the role of cell surface Transmembrane Kinase (TMK) in the transcriptional auxin-regulated signalling that is responsible for apical hook development in *Arabidopsis*. This TIR1-independent pathway involves auxin-triggered cleavage of TMK, followed by binding of TMK to auxin-responsive proteins IAA32 and IAA34 which are stabilized and act as transcriptional repressors.

## 5. Development

This session involved 18 oral contributions. These covered different aspects of the hormonal control of plant development, including shoot apical meristem maintenance, cell and organ polarity establishment, early leaf morphogenesis, provascular cell proliferation, cambial activity, protophloem specification, phyllotactic patterning, control of the lateral root angle, panicle and embryo development, and trichome formation. Bert De Rybel (VIB-Ghent, Belgium) presented data on the hormonal control of oriented cell division instructive for organ shaping. The identification of cytokinin biosynthetic genes *LOG4* and *LOG3* as direct targets of the TMO5/LHW (TARGET OF MONOPTEROS5/LONESOME HIGHWAY) dimer complex indicates that cytokinin biosynthesis plays a crucial role in cell divisions preceding vascular tissue formation. Novel transcription factors were found which control specific TMO5/LHW downstream responses leading to oriented cell divisions during vascular development. Jan Lohmann (Heidelberg University, Germany) showed the role of transcription factor WUSCHEL in the gating of auxin response in the shoot apical meristem stem cells. Christian Hardtke (University of Lausanne, Switzerland) introduced a model of the molecular rheostat responsible for the specification of root protophloem sieve elements. This machinery involves proteins BRX (BREVIS RADIX) and AGC family protein kinase PAX (PROTEIN KINASE ASSOCIATED WITH BRX), which are under the control of the auxin gradient and co-localize with auxin efflux carrier PIN1 at the root-ward plasma membrane of developing protophloem cells. Lars Ostergaard (JIC, Norwich, UK) focused on the identification of the molecular machinery of a recently described alternative auxin signalling pathway. This pathway is mediated by the auxin response factor ETTIN, which plays a particularly important role during the establishment of polarity in organ development. Laila Moubayidin (JIC, Norwich, UK) described a transcriptional network controlling bilateral to radial development in the *Arabidopsis* gynoecium. This network is responsible for the generation of the radially symmetric ring of auxin-responsive cells as well as restriction of sensitivity to cytokinins, allowing the development of a radial style. Victoria Mironova (Novosibirsk State University, Russia) introduced a systematic microscopy-based analysis of cell division activities in the root of *Arabidopsis* along the division and transient zone. By this approach, auxin and cytokinin biosynthesis mutants were compared, and the inhibitory effect of cytokinins on anticlinal cell divisions in the stele was uncovered. Helene Robert Boisivon (CEITEC, Czech Republic), who recently showed that maternal tissues coordinate seed development by supplying auxin to the early embryo from the integuments of the ovule, focused on regulators of auxin biosynthetic genes from the L-AFL protein network. Jürgen Kleine-Vehn (BOKU, Austria) presented his results on GWAS (Genome-Wide Association Studies) analysis of changes in the angle of the lateral root. The role of cytokinins in the determination of the angle of lateral root outgrowth is implicated by up-regulation of cytokinin signalling as well as by metabolic changes. Asymmetric cytokinin signalling counterbalances in lateral roots’ auxin-dependent gravitropic growth. Teng Zhang (University of Helsinki, Finland) showed how a phyllotactic patterning is established in Asteraceae flower heads. Using micro-CT imaging, growth dynamics during the early ontogeny of flower heads were recorded in the Asteraceae family. It was shown that the Fibonacci order cannot be formed de novo and that patterning is species-specific and maintained by auxin gradients. Leo Serra (INRA, CNRS, France) described patterning processes during early leaf morphogenesis and the role of CUC (CUP-SHAPED COTYLEDON) transcription factors and auxin response. As shown by time-lapse imaging of leaf serration development in reporter lines, one of the CUC genes is needed for the local repression of cell growth through lowering auxin response at the tip of the developing serration. Eric Schaller (Dartmouth College, USA) provided an overview of the role of type-B RRs in the control of gene expression within the genome-wide context. Binding of one of the type-B RRs, ARR10, was shown to be positively controlled by cytokinins for both cytokinin-activated and repressed genes. Surprisingly, ARR1, ARR10, and ARR12 seem to be necessary for root formation in hypocotyl explants. The subnetwork mediating cytokinin action during different stages of panicle development was described. In rice, type-B RRs were shown to be involved in trichome formation. Rishikesh Bhalerao (Umeå University, Sweden) pointed to the central role of the cell wall in the integration of hormonal signals regulating the rate of cell elongation. He showed that the xyloglucan composition of the cell wall determines the formation of the *Arabidopsis* apical hook, influencing, among other things, the differential localization of auxin carriers. Wendy Peer (University of Maryland, USA) presented the identification of the second copy of the gene for the IAA oxidizing enzyme, DAO2. The function of DAO2 was shown to be independent of DAO1, which regulates floral organ development. Melis Kucukoglu (University of Helsinki, Finland) reported on the role of cytokinins in the control of cambial activity in poplar. Targets of cytokinin action in the control of cambial activity included peptide hormones and specific transcription factors. Interestingly, the cytokinin-regulated genes largely overlapped with cambium-specific genes, further substantiating the role of cytokinins as important regulators of cambial activity. John Vaughan-Hirsch (University of Nottingham, UK) summarized the role of phosphotransfer proteins, both those containing the conserved His and those having the phosphoaccepting His replaced by Gln, in the root patterning of rice.

## 6. Transport

In this session, seven oral contributions were presented demonstrating new observations on the regulation of auxin transport. In particular, key findings regarding PIN- and ABCB-facilitated auxin transport, as well as recent data on the indole-3-butyric acid (IBA) transporter, were shown. Markus Geisler (University of Fribourg, Switzerland) showed that FKBP42/TWD1 (FK506-binding protein/TWISTED DWARF1) regulates the polar auxin distribution via interaction with ABCB-type (ATP Binding Cassette Subclass B) ABC transporters modulating their subcellular trafficking. Cytoplasmic HSP90 isoforms were identified as interacting partners of TWD1. ABCB transporters are thus dependent on cytoplasmic HSP90 proteins as well as on FKBP42/TWD1 activity, stabilizing their localization in the plasma membrane and affecting auxin transport and, consequently, plant development. Matous Glanc (IST, Austria), from Jiří Friml’s group, focused on the asymmetric subcellular distribution of PIN auxin efflux carriers. PIN2 polarity establishment was shown to be directly dependent on endocytosis but not requiring a functional cytoskeleton. Lucia Strader (Washington University in St. Louis, USA) described the identification of TRANSPORTER OF IBA1 (TOB1) that limits lateral root formation, likely by sequestering IBA to the vacuole, preventing its conversion to the active auxin pool. IBA transporter TOB1 accumulates in response to cytokinins, linking cytokinin and auxin functions in the regulation of lateral root formation. Illas El Houari (VIB-UGent, Belgium) presented a new tool for the study of auxin cellular and tissue transport: a novel natural auxin efflux inhibitor, *cis*-cinnamic acid. Angus Murphy (University of Maryland, USA) summarized the current knowledge on ABCB transporters. Long-distance polar auxin transport depends on the presence of these auxin transporters, generally associated with multisubstrate specificity and the exclusion of hydrophobic anions from plasma membrane leaflets. ABCB exclusion mechanisms are important for limiting polar auxin streams to the central vascular cylinder of hypocotyls and roots. The following two talks were focused on the regulation of PIN transporter activity. Claus Schwechheimer (Technische Universität München, Germany) demonstrated how PIN protein phosphorylation by protein kinases, such as the D6 PROTEIN KINASE (D6PK), promotes their auxin efflux carrier activity. Ivan Kashkan (CEITEC, Masaryk University, Czech Republic) showed that alternative splicing of PIN auxin efflux carriers might be responsible for their distinct trafficking properties.

## 7. Interactions and Crosstalk

In this session, 11 oral contributions covering recent knowledge on the interactions among auxins, cytokinins, strigolactones, and jasmonates was presented. Crosstalk between plant hormones and abiotic factors (e.g., light or boron) was reported as well. Ottoline Leyser (University of Cambridge, UK) summarized recent knowledge on the crosstalk between auxin, cytokinins, and strigolactones during shoot branching. The dual mode of branching control, consisting of mutually largely independent mechanisms, i.e., carrier-driven generation of auxin gradients and local expression of transcription factor BRC1, was elucidated. These two mechanisms are under the control of both strigolactones and cytokinins, providing robustness to the regulation of branching. Abdellah Lakehal (Umeå University, Sweden) reported on the downstream targets of jasmonate signalling during adventitious root formation. Potential crosstalk with both auxin and cytokinin pathways has been suggested. Anthony Bishopp (University of Nottingham, UK) presented a multiscale mathematical modelling approach for the vascular tissue formation in plants with more complex vasculature patterning. The pattern does not seem to fully depend on the asymmetric distribution of auxin and cytokinins, but it rather relies on spatial constraints and growth. The presented model could potentially explain the large diversity of vasculature formation in flowering plants. Hagai Yasuor (Gilat Research Center, Izrael) described complex hormonal interactions, predominantly of auxins and cytokinins, during flower and fruit development at different developmental stages in tomato. Eva Benková (IST, Austria) focused her talk on the synergistic crosstalk between auxin and cytokinins. She presented identification of a novel player, protein SYAC1 (SYnergistic Auxin Cytokinin 1). The up-regulation of SYAC1 is strictly dependent on both auxin and cytokinin, and the protein acts as the developmentally specific regulator of the cell secretory pathway necessary for cell wall modifications during cell elongation. Tereza Dobisová (CEITEC, Czech Republic) presented data on cytokinin–light crosstalk. The Phytochrome A regulated transcription factors PHYTOCHROME INTERACTING FACTOR 3 (PIF3) and CIRCADIAN CLOCK ASSOCIATED 1 (CCA1) were found to bind to the promoter of sensor histidine kinase CKI1, which seems to be involved in the control of root growth, hypocotyl elongation, and hook formation during skotomorphogenesis. The light-induced CKI1 seem to regulate multistep phosphorelay signalling, thus mediating light-dependent control of sensitivity to cytokinins. Michal Karady (Umeå University, Sweden) introduced a second generation of the organic electronic ion pump. This device has been optimized for the accurate application of physiologically relevant amounts of various phytohormones and growth substances to individual growth zones of *Arabidopsis* root, which opens new opportunities to study hormonal crosstalk. Michaela Sylvia Matthes (University of Missouri, USA) demonstrated the functions of boron in plants. This element is known to stabilize pectins within the cell wall. Boron deficiency in maize was found to reduce meristem size and affect the number of auxin- and cytokinin-regulated proteins, including dramatic de-localization of the maize PIN1 homologue.

## 8. Interaction with the Environment

The session, containing 14 lectures, was opened by Francois Barbier (The University of Queensland, Australia) from the lab of Christine Beveridge. He reported on the regulation of shoot branching after decapitation, which seems to be governed by sugar as well as by the phytohormones cytokinins and strigolactones. He mentioned a poor correlation between the initial bud release and auxin depletion from the stem after decapitation. In contrast, the onset of bud release correlated well with sucrose mobility in plants and its accumulation in buds which was able to trigger bud outgrowth. The depletion of sugars from axillary buds decreased bud release even at conducive hormone levels. The sugar signalling involved trehalose 6-phosphate. Anne Cortleven (Freie Universität Berlin, Germany) showed that prolongation of the light period at the expense of the dark period caused a novel type of abiotic stress—altered photoperiod stress. That type of stress involved oxidative burst (as indicated by up-regulation of catalase and apoplastic peroxidase activities) as well as increased jasmonic acid concentration. High susceptibility of cytokinin-deficient plants to this kind of stress indicates intensive crosstalk of the cytokinins with the circadian clock, jasmonic acid pathway, and the oxidative stress response. Aaron Rashotte (Auburn University, USA) reported on the role of cytokinin response factors (CRFs) in two abiotic stresses—salinity and the oxidative one. The former stress was associated with cytokinin up-regulation, while the latter one with their down-regulation. Under salt stress, CRF2 was the main regulator, whereas the regulators under oxidative stress were CRF5 and CRF6. Thomas Roitsch (University of Copenhagen, Denmark) showed that cytokinins trigger resistance against some of the virulent hemibiotrophic pathogens, e.g., *Pseudomonas syringae*, in tobacco plants. This cytokinin-triggered resistance correlated with production of antimicrobial phytoalexins as well as with activation of the salicylic acid defence network. Benoit Lacombe (CNRS, INRA, France) is an expert on nitrate signalling, which is mediated by nitrate transporter NRT1.1/NPF6.3. This membrane protein exhibits nitrate-dependent auxin transport capacity. The macromolecular complex, regulating both nitrate transport and sensing, involves a part of NRT1.1/NPF6.3, along with CIPK23, a protein kinase, and CBL9, a calcium sensor, and protein phosphatase. Systemic nitrate response is also affected by cytokinins. Branka Salopek-Sondi (Ruđer Bošković Institute, Croatia) compared hormonal responses to drought stress in selected Brassica varieties (Chinese cabbage, white cabbage, and kale). A higher increase in the levels of abscisic acid and jasmonates was obtained in more sensitive varieties, while more tolerant kale exhibited a more profound increase in salicylic acid, *cis*-zeatin(riboside), and auxin. Maria Savina (Institute of Cytology and Genetics SB RAS, Russian Federation) reported that cold stress caused the programmed death of columella stem cell daughters (CSCDs) in *Arabidopsis* roots, which allowed them to sustain the auxin maximum in the quiescent centre. This local auxin maximum helps the root to withstand stress and to recover faster. Jutta Ludwig-Müller (Technische Universität Dresden, Germany) described the up-regulation of four *GH3* genes encoding auxin conjugate synthetases in male flower organs and gametophytes during heat stress, which resulted in inhibition of pollen development, anther release, and pollen viability correlating with a reduction of the fruit set. Marta Del Bianco (University of Leeds, UK) presented a mathematical model for the regulation of the gravitropic behavior of *Arabidopsis* roots. The model provides a mechanistic interconnection of the three main gravitropism hypotheses: the starch statolith, the auxin asymmetric accumulation, and the angle-dependent stimulation. Cris Argueso (Colorado State University, USA) described the priming effect of cytokinins against biotrophic pathogens. Cytokinin action includes chromatin regulation, which may represent a new mechanism of defence priming against biotic stresses.

## 9. Conclusions

The symposium was very informative, and both oral and poster presentations covered valuable, newly published data as well as overviews of the progress made in specific topics of hormone studies. Some new results have been included in this Special Issue of *IJMS*. Moreover, we are sure that many other presented data will become a basis for new, exciting, original reports in the near future. In general, the ACPD 2018 symposium illustrated the significance of the progress in the state of the art, especially in the hormone mode of actions and the complex crosstalk in development regulation and environmental interactions. Great progress has been made in elucidating the role of hormonal regulations in the control of gene expression, frequently of a dual (both positive and negative) nature. In this respect, the role of plant hormones in various types of epigenetic regulations is among the emerging hot topics. In parallel, understanding the role of protein structure, specific protein–protein interactions, and the formation of (higher-order) protein complexes has become another clear focus of modern plant biology necessary to decipher the complex hormonal regulations of plant development.

A very valuable part of the meeting included stimulating discussions among people of different areas of expertise. The free flow of information contributed to intensive interaction between scientists and students who participated in substantial numbers in this meeting. This platform is a very good starting point for the next ACPD 2022 symposium, which we all are looking forward to.

